# From the Nares to the Bone Marrow: A Role for Transarterial Embolization in an Aberrant Life-Threatening Cause of Epistaxis

**DOI:** 10.7759/cureus.31278

**Published:** 2022-11-09

**Authors:** Andrew Engel-Rodriguez, Natalie Engel-Rodriguez, Mariola A Vazquez Martinez, William Cáceres-Perkins, Juan Vazquez-Fuster, Miguel Anzalota-Del Toro, Charmaine Perez-Del Valle

**Affiliations:** 1 Internal Medicine, VA Caribbean Healthcare Systems, San Juan, PRI; 2 Internal Medicine, San Juan Bautista School of Medicine, Caguas, PRI; 3 Hematology and Oncology, San Juan City Hospital, San Juan, PRI; 4 Hematology and Oncology, VA Caribbean Healthcare Systems, San Juan, PRI; 5 Cardiology, VA Caribbean Healthcare Systems, San Juan, PRI

**Keywords:** transarterial embolization, life threatening, facial artery, maxillary artery, plasma cell dyscrasia, management, hemorrhage, embolization, epistaxis, multiple myeloma

## Abstract

Identifying underlying bleeding diathesis that is amenable to medical therapy must be determined to provide timely treatment and minimize morbidity. Nasal bleeding is viewed as an annoyance by most who suffer from its episodes. However, it can at times be a baleful ailment that can compromise a patient's airway, breathing, and circulation, which can result in death. A 75-year-old Hispanic man presented with life-threatening epistaxis and was ultimately diagnosed with multiple myeloma (MM). The patient suffered profuse bleeding and hemodynamic compromise, requiring endoscopic nasal packing, red cell transfusions, platelet transfusions, and right external carotid artery angiogram with maxillary arteries embolization prior to chemotherapy. Embolization of maxillary arteries helped to stabilize the patient to diagnose MM and start definitive management with chemotherapy. On data review, we could not find another case with severe epistaxis secondary to MM, which was controlled with endovascular embolization. This case highlights the difficulties in managing a rare condition and the importance of a multidisciplinary approach in patients who present with life-threatening epistaxis secondary to plasma cell dyscrasia.

## Introduction

Almost 10% of all hematologic malignancies are accounted for by multiple myeloma (MM) [1]. It is described by the excessive production of structurally homogeneous immunoglobulins secondary to the proliferation of plasma cells in the bone marrow [1]. The criteria for the diagnosis of active MM as per the National Comprehensive Cancer Network includes the presence of biopsy-confirmed bony or extramedullary plasmacytoma, clonal bone marrow plasma cells ≥ 10%, and one or more special characteristics associated with MM [1]. These are known as myeloma-defining events, and they are hypercalcemia, renal failure, anemia, MRI scan with evidence of one or more focal ≥ 5 mm lesions, skeletal radiography with one or more osteolytic bone lesions, abnormal serum-free light chain (FLC) < 0.01 (involved lambda), or ratio ≥ 100 (involved Kappa) [1]. Hypercalcemia, renal failure, anemia, and bone pains are the most common findings at presentation [1]. Epistaxis is a common complaint in patients requiring evaluation by an otorhinolaryngologist [2]. Most nose bleeds are self-limiting, spontaneous, and benign, but some can be recurrent. On the nasal septum, you find the Little’s area, which is where the Kiesselbach plexus forms. More than 90% of bleeds arise from Little's area [2]. According to the available medical literature, 70% to 80% of adulthood epistaxis is idiopathic [2]. However, the true prevalence of epistaxis is unknown because most episodes are not reported [3]. MM presenting with epistaxis is rare; hence, MM as the etiologic factor is seldom considered [4]. We herein report a case of a patient diagnosed with MM that initially manifested as a life-threatening nasal bleed; also, we review the association between MM and the tendency of hemorrhage.

## Case presentation

**Figure 1:**
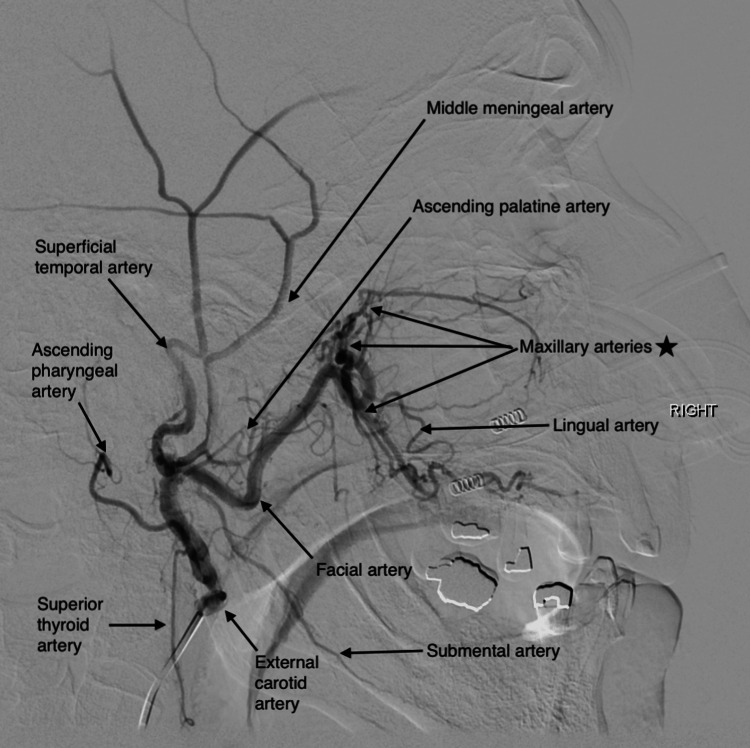
External carotid artery angiogram Right side lateral view of external carotid artery angiogram before sub-selective distal right facial artery and maxillary arteries embolization. (black star). The patient is a case of type 2 facial artery anatomical variant.

**Figure 2:**
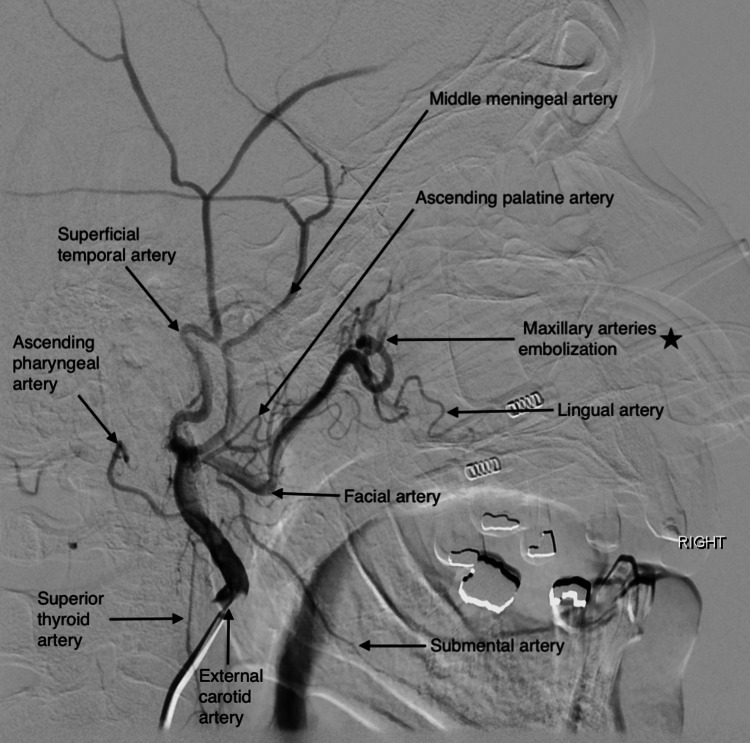
External carotid artery angiogram after embolization of distal facial artery and maxillary arteries Right side lateral view of external carotid artery angiogram after sub-selective distal right facial artery and maxillary arteries embolization. (black star). The patient is a case of type 2 facial artery anatomical variant.

A 75-year-old Hispanic male patient with a medical history of large cell lymphoma treated with R-CHOP treatment (rituximab, cyclophosphamide, doxorubicin hydrochloride, vincristine sulfate, and prednisone) in 2012, benign prostatic hypertrophy, essential hypertension, and type 2 diabetes mellitus was sent from primary care clinics to the Emergency Room (ER) due to marked decrease in hemoglobin from 15 g/dL to 6.3 g/dL, intermittent episodes of epistaxis, weakness, and fatigue of one-month duration. At ER, he developed active epistaxis and underwent nasal endoscopy with nasal packing on the right nostril. He denied any bone pain, joint pain, easy bruising, petechiae, spontaneous hemarthrosis, or rashes. The initial labs were remarkable for pancytopenia, elevated total protein with albumin/globulin dissociation, acute kidney injury (KDIGO [Kidney Disease: Improving Global Outcomes] grade 3), normal calcium level, urinalysis with proteinuria, prolonged PT (prothrombin time), normal INR/PTT (international normalized ratio/partial thromboplastin time), and significant Rouleaux formation on peripheral blood smear (Table 1). Despite nasal packing, the patient required 8 packed red cell transfusions over the course of four days and was transferred to the medical intensive care unit for close hemodynamic monitoring due to non-resolving epistaxis and hemodynamic instability. Sub-selective right facial artery angiography (Figure 1) and embolization of the right maxillary arteries were performed (Figure 2).

**Table 1:** Initial and specialized labs INR, international normalized ratio; UPEP, urine protein electrophoresis; SPEP, serum protein electrophoresis; COL/EPI, collagen/epinephrine coated platelet membranes; COL/ADP, collagen/adenosine diphosphate coated platelet membranes **Altered lab value

Lab test	Lab value	Lab range
Initial labs		
Hemoglobin**	6.3 g/dL	12.6-17.8 g/dL
White blood cells**	2.6 L x 10-3/uL	4.3-9.3 L x 10-3/uL
Platelets**	94 L x 10-3/uL	155-371 L x 10-3/uL
Serum total proteins**	10.9 g/dl	6-8.5 g/dL
Albumin	2.8 g/dL	2.6-5.2 g/dL
Globulin**	8.1 g/dL	1.5-3.5 g/dL
Prothrombin	16.1 s	11.8-15s
Partial thromboplastin time	37 s	22-38s
INR	1.29	
Creatinine**	4.6 mg/dL	0.7-1.5 mg/dL
Calcium	9.0 mg/dL	8.5-10-5 mg/dL
Urine analysis**	Protein: 100 mg/dL	Negative
Specialized labs		
total immunoglobulin A**	3688 mg/dL	131-407 mg/dL
total immunoglobulin G	180 mg/dL	744-1660 mg/dL
Total immunoglobulin M	13.52 mg/dL	20-220 mg/dL
UPEP spot urine**	M-spike in urine, Kappa	
SPEP**	M-spike 3.7 g/dL beta region	
Kappa light chain**	1114 mg/L	3.3-19.4 mg/L
Lambda light chain**	8.9 mg/L	5.7-26.3 mg/L
Kappa/lambda ratio**	125	0.26-1.65
B2 microglobulin**	29.13 mg/L	0.7-3.4 mg/L
Factor X	87	70-120
COL/EPI**	>300 s	81-154 s
COL/ADP**	288 s	53-105 s

**Figure 3:**
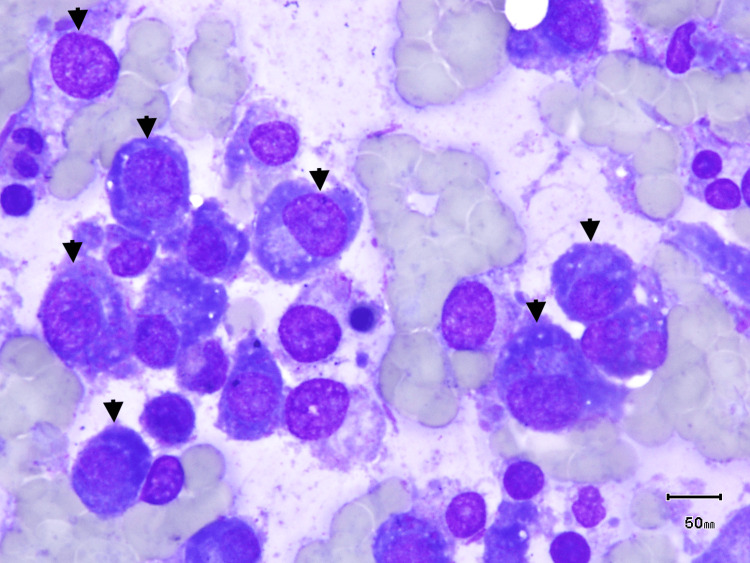
Bone marrow aspiration Bone marrow aspirate smear with many plasma cells (black arrowheads).

Myelodysplastic syndrome as the cause of his pancytopenia had to be considered in the differential given the patient's history of large B-cell lymphoma treated with chemotherapy. A PET/CT was performed, which demonstrated no abnormal hypermetabolic lymphadenopathy. Bone marrow biopsy revealed elevated plasma cells at 43.4% (Figure 3). Serum protein electrophoresis revealed a monoclonal (M) spike IgA Kappa of 3.7 g/dL. Immunofixation disclosed monoclonal IgA kappa. Free kappa/lambda (K/L) ratio was elevated at 125 with 1,114 total kappa chains. In urine protein electrophoresis, free kappa light chains were detected.. COL/EPI and COL/ADP are severely prolonged, indicating possible platelet dysfunction. Factor X levels were within reference ranges. Due to elevated plasma cells (>10%), altered renal function (CrCl <40 mL/min or serum creatinine >2 mg/dL), and a K/L ratio > 100 (K/L: 125.22), the diagnosis of MM was confirmed. B2 microglobulin was elevated at >5.5 (Table 1), signaling stage 3 MM as per the international staging system. Cytogenetics revealed extra copy of lq23, IGH/FGFR rearranged, hyperdiploid, and monosomy 13, which entails high-risk MM. The patient was started on CyborD treatment (bortezomib, cyclophosphamide, and dexamethasone) and had no further episodes of hemorrhage. His renal function is improving, as well as his pancytopenia.

## Discussion

MM commonly presents with a wide variety of symptoms, which are known as CRAB symptoms [4]. The “C” stands for hypercalcemia, which is seen in approximately 13% of cases, “R” stands for renal failure, which is seen in approximately 20%-40% of cases, “A” stands for anemia, which is seen in approximately 70% of cases, and “B” stands for lytic bone lesions, which are seen in approximately 80% of cases [4]. Other common MM manifestations are weight loss and recurrent infections [4]. The patient we report had renal failure and anemia on presentation; however, these abnormal laboratory results were originally attributed to blood loss and resulting hypoperfusion. Our reasoning was that the anemia was secondary to the epistaxis and the renal failure was secondary to hypoperfusion due to anemia. MM was not in the differential on admission. Now the patient also had pancytopenia in initial laboratories, and therefore a peripheral blood smear was sent to better characterize the abnormality. This and the albumin/globulin dissociation were the initial clues that prompted us to start looking at MM as the underlying cause of the disease. Rouleaux formations were identified on the peripheral blood smear, and once the patient was hemodynamically stable, he was taken for a bone marrow biopsy.

In plasma cell dyscrasias, abnormal coagulation tests are commonly seen. Despite this, clinically significant bleeding complications are seldom present [5]. Several retrospective studies on MM did not report bleeding as a presenting symptom [5]. While epistaxis may often only be considered an annoyance by people who suffer episodes, it is important to be aware that it can rarely herald an underlying sinister pathology, hence warranting further evaluation. Numerous processes have been presumed to account for hemorrhage seen in plasma cell dyscrasia. These include coagulation disorders of both secondary and primary hemostasis. M-protein is a monoclonal paraprotein produced by the malignant plasma cells, which can bind to coagulation factors, platelets, von-Willebrand factor, and fibrinogen [6]. Other causes are tissue injury secondary amyloid accumulation causing protein precipitation on blood vessel walls, thrombocytopenia, hyperviscosity, circulating heparin-like anticoagulant, factor X deficiency, acquired von Willebrand syndrome, and vascular endothelium damage [7-9]. In our patient, the pathology causing nose hemorrhage is thought to be multifactorial. We understand that protein precipitation on blood vessel walls had a role in precipitating the uncontrolled bleeding given the markedly elevated total protein. The patient also had thrombocytopenia and concomitant platelet dysfunction due to renal failure as evidenced by prolonged COL/EPI and COL/ADP predisposing the patient to severe bleeding episodes.

Randomized trials to investigate distinct management approaches and their outcomes for life-threatening bleeding diathesis secondary to MM are nonexistent. Trials to investigate are likely difficult as bleeding diathesis in MM is largely multifactorial and incidence is low. As such, most current recommendations are based on small case series or anecdotal evidence. Our patient received symptomatic treatment with endoscopic nasal packing, packed red blood cell transfusions, platelet transfusions, and endovascular embolization. Embolization of the right maxillary artery stabilized his condition, giving us time to diagnose MM and initiate treatment with CyborD promptly. On data review, we could not find another case in which life-threatening epistaxis secondary to MM was managed with embolization of the maxillary arteries. It is important to note that embolization helped bridge the patient into definitive management of MM, the underlying cause of his bleeding diathesis, with chemotherapy. Though symptomatic management strategies can temporarily improve the patient's situation, the mainstay of definitive management is always treating the underlying disease. Response to therapy was demonstrated with improved renal function, abortion of nasal hemorrhages, improvement of pancytopenia, decreasing numbers of abnormal paraprotein, and a decrease in total protein levels, a factor implicated in hyperviscosity syndrome. This case is interesting given the uncommon presentation of MM with a life-threatening bleed and successful unconventional management with embolization.

## Conclusions

In most cases, the cause of epistaxis is unknown, and the bleeding is self-limiting; however, it can infrequently herald a much baleful cause. Life-threatening epistaxis secondary to MM is rare, and management is challenging because of the multifactorial pathogenesis and the undiagnosed acquired disorders. Even though the incidence of life-threatening epistaxis/bleeding in MM is rare, clinicians should pay attention to laboratory abnormalities and have suspicion of the possibility of MM in those settings. Randomized trials to investigate distinct management approaches to life-threatening bleeding diathesis secondary to MM and their outcomes are nonexistent. This is likely due to the low incidence of the manifestation. Most of the current management recommendations are based on small case series or anecdotal evidence. This case is special because on literature review we were unable to find another case of life-threatening epistaxis secondary to MM that was managed with endovascular embolization. Before diagnosing MM, our patient's life-threatening epistaxis was managed with nasal packing, packed red cell transfusions, platelet transfusions, and maxillary arteries embolization. He had positive outcomes following the procedure without any significant complications. These interventions helped bridge the patient into definitive management of MM, with chemotherapy, and no further episodes of nasal hemorrhage have occurred.

## References

[1] Kumar SK, Callander NS, Hillengass J (2019). NCCN guidelines insights: multiple ,yeloma, version 1.2020. J Natl Compr Canc Netw.

[2] Padgham N (1990). Epistaxis: anatomical and clinical correlates. J Laryngol Otol.

[3] Douglas R, Wormald PJ (2007). Update on epistaxis. Curr Opin Otolaryngol Head Neck Surg.

[4] Iwaniec T, Zdziarska J, Jurczyszyn A (2019). Abnormal hemostasis screening tests leading to diagnosis of multiple myeloma. Acta Haematologica Polonica.

[5] Kyle RA, Gertz MA, Witzig TE (2003). Review of 1027 patients with newly diagnosed multiple myeloma. Mayo Clin Proc.

[6] Coppola A, Tufano A, Di Capua M, Franchini M (2011). Bleeding and thrombosis in multiple myeloma and related plasma cell disorders. Semin Thromb Hemost.

[7] Saif MW, Allegra CJ, Greenberg B (2001). Bleeding diathesis in multiple myeloma. J Hematother Stem Cell Res.

[8] Eby C (2009). Pathogenesis and management of bleeding and thrombosis in plasma cell dyscrasias. Br J Haematol.

[9] Rahman S, Veeraballi S, Chan KH, Shaaban HS (2021). Bleeding diathesis in multiple myeloma: a rare presentation of a dreadful emergency with management nightmare. Cureus.

